# Editorial: Recent Advances in Flowering Time Control

**DOI:** 10.3389/fpls.2016.02011

**Published:** 2017-01-05

**Authors:** Christian Jung, Klaus Pillen, Dorothee Staiger, George Coupland, Maria von Korff

**Affiliations:** ^1^Plant Breeding Institute, Christian-Albrechts-University of KielKiel, Germany; ^2^Plant Breeding Institute, Martin Luther University of Halle-WittenbergHalle, Germany; ^3^Molecular Cell Physiology, Faculty of Biology, Bielefeld UniversityBielefeld, Germany; ^4^Department of Plant Developmental Biology, Max Planck Institute for Plant Breeding ResearchCologne, Germany; ^5^Cluster of Excellence in Plant Sciences, Heinrich-Heine-University DüsseldorfDüsseldorf, Germany

**Keywords:** floral transition, crop plants, Arabidopsis, phenological development, yield, evolution

The phenological development of plants can be broadly divided into 4 stages, embryo/juvenile, adult (all vegetative stages), reproductive (the generative stage), and senescent. This research topic focusses on the transition from vegetative growth to reproductive development, commonly referred to as floral transition. Plants have coordinated the seasonal timing of flowering and reproduction with the prevailing environmental conditions. In agriculture, flowering is a prerequisite for crop production whenever seeds or fruits are harvested. In contrast, avoidance of flowering is necessary for harvesting vegetative parts of a plant such as tubers or roots. Late flowering also severely hampers breeding success due to long generation times. Thus, flowering time regulation is of utmost importance for genetic improvement of crops.

In the past decades, we have gained increasing knowledge of flowering time regulation in model species such as *Arabidopsis thaliana* (Blümel et al., 2015). Genes coordinately regulating floral transition have been grouped into different pathways that have recently been illustrated in a WIKIPATHWAYS web interface (http://wikipathways.org//index.php?query=flowering&title=Special%3ASearchPathways&doSearch=1&sa=Search). Several of these pathways are activated by different environmental factors such as low temperature, day length, light intensity, or stress. Conservation of major flowering-time regulators and regulatory pathways between different species as well as increased availability of genome sequences and improvements in computational biology offer a unique opportunity to study flowering time genes across species. In general terms, the central elements that perceive day-length signals to control floral transition are conserved across the plant kingdom. *CONSTANS* (*CO*)-like genes and PHOSPHATIDYLETHANOLAMINE-BINDING PROTEIN (PEBP) encoding genes play major roles in these pathways and were first identified by genetic analysis in Arabidopsis. *CO*-like sequences seem to exist in all plants. Recent evidence indicates that *CO* of Arabidopsis arose from a family-specific duplication and similar events might have occurred independently in many plant families (Simon et al., 2015). In contrast, plants from the Amaranthaceae family are lacking a true *CO* ortholog (Dally et al., 2014).

This research topic is focused on flowering time control in cultivated species. It contains nine review, perspective, and opinion articles and 14 original research articles which cover a large range of organisms from model species to crops.

New components have been added to the network of flowering time regulators mostly working upstream of key regulator elements, e.g., GATA transcription factors, small RNAs, in particular microRNAs (miRNAs) or sugar molecules. The GATA transcription factors *GATA, NITRATE-INDUCIBLE, CARBON-METABOLISM INVOLVED* (*GCN*), and *GCN-like* (*GNL*) previously identified as growth regulators mediating control by several phytohormones have emerged as repressors of flowering, acting via *SOC1* (Richter et al., 2013; Behringer and Schwechheimer). During the juvenile to adult phase transition, a gradual decrease in miR156 and a reciprocal increase in miR172 ultimately leads to the activation of *FLOWERING LOCUS T* (*FT*) (Wang et al., 2009a; Wu et al., 2009). To facilitate the genome-wide analysis of small RNA-seq data, the DARIO tool developed for animals has been adapted for use in plants (plantDARIO) (Patra et al.).

The research topic also reflects the immense technical progress from the past years. Initially, flowering time regulators from crops were mainly cloned due to their sequence homology with known flowering time genes, mainly from Arabidopsis although the *INDETERMINATE* flowering gene was cloned from maize by transposon tagging (Colasanti et al., 1998). Later, new genes were identified from crops using flowering time QTLs (quantitative trait loci) by map-based cloning approaches. Now, whole genome or candidate gene association mapping and transcriptome analysis have become important strategies (Schiessl et al.). For instance, whole transcriptome analysis revealed the circadian clock homolog of EARLY FLOWERING 3 (ELF3) and mapping-by-sequencing applied on exome-capture data from phenotypic bulks identified *PHYTOCHROME C* as important components of photoperiodic flowering in barley (Faure et al., 2012; Pankin et al., 2014). In addition, the wild barley nested association mapping population HEB-25 was used to associate major flowering time genes with phenological development in different field environments (Maurer et al., 2015, 2016). Furthermore, several flowering time genes like *Ppd-H1* (*PRR37*) and *HvSDW1* (*GA20ox2*) were linked to both, developmental and yield-related traits. In a salinity tolerance study with HEB-25, the wild barley allele at the *HvCEN* locus (Antirrhinum *CENTRORADIALIS, TFL1*-like) promoted flowering and maturity, resulting in a higher harvest index and a higher yield under salt stress in the field (Saade et al., 2016). These findings indicate that searching for allelic variants of known flowering time genes, also taken from related wild species, may substantially support future plant breeding efforts to increase plant performance under optimal cultivation conditions as well as under stress.

Flowering time regulation is strongly conserved among the Brassicas to which Arabidopsis also belongs. Two articles (Guo et al.; Schiessl et al.) describe flowering time genes from oilseed rape where the vernalization pathway with its central element *FLOWERING LOCUS C* (*FLC*) is essentially the same as in Arabidopsis. In contrast, an *FLC* homolog from beet was proven not to be a major regulator of vernalization response in biennial beets Vogt et al. New sequence variation has been induced in rapeseed by EMS mutagenesis which gave rise to plants with altered flowering time in spite of the polyploid nature of this species. Mutations within a single gene can have a big impact on flowering time even if there are several paralogs of an Arabidopsis flowering time gene present in the rapeseed genome.

The research topic demonstrates that the range of model species has been constantly increased to allow a broader range of flowering-related traits to be studied. *Arabis alpina* and *Brachypodium distachyon* serve as models for perennials (Wang et al., 2009b) and for grasses Woods et al., respectively. A recent overview on flowering regulation in grass species is given in this research topic (Fjellheim et al.). The authors discuss molecular pathways that control seasonal flowering responses in the *Pooideae* sub-family and how variations in flowering time gene activities contributed to the adaptation to different environments. Refined flowering time regulatory pathways have been identified from rice (Shrestha et al., 2014) and barley/wheat (Chen et al., 2014) (Mulki and von Korff, 2016). Loscos et al. show that natural allelic variation in copy number of the florigen *HvFT1* is present in European spring barley cultivars lacking a vernalization requirement to initiate flowering (Loscos et al., 2014). However, no clear relationship between *HvFT1* copy number and expression was observed in a set of diverse spring barley genotypes.

Some articles from this research topic highlight multiple functions of flowering time genes beyond floral transition. These genes impact multiple developmental processes and they are regulators of yield components and stress tolerance (Kazan and Lyons, 2016). In this respect, members of the PHOSPHATIDYLETHANOLAMINE-BINDING PROTEIN (PEBP) gene family, such as *FLOWERING LOCUS T* (*FT*) and *TERMINAL FLOWER 1* (*TFL*) of Arabidopsis attracted the highest attention. As highlighted for tomato (Lifschitz et al.) and for rice (Izawa et al., 2016) fine tuning of the *SFT/SP* (which are true orthologs of *FT* and *TFL*) ratio is an important process for patterning plant architecture. Lifschitz et al. explain how the ratios between *FT*-like and *TFL1*-like genes control the patterning of the shoot systems across many different plants. In tomato, an increase of the florigen protein *SINGLE FLOWER TRUSS* (*SFT, FT*-like), relative to the anti-florigen protein *SELF PRUNING* (*SP, TFL1*-like) induces growth arrest and termination of meristems across the tomato shoot, while high relative levels of *SP* promote the formation of an indeterminate vegetative inflorescence. Consequently, *SFT/SP* ratios determine the number of flowers and eventually, tomato fruits per inflorescence. Naturally occurring mutations have been selected in both gene families to adapt crops to different environments and to increase productivity. *TFL1* mutants have been frequently used in breeding (e.g., tomato, soybean, roses, and barley). Likewise, mutations of *FT*-like genes were selected in sunflower, sugar beet, rice, potato, and wheat. Moreover, single point mutations within *FT-* and *TFL1* orthologs can drastically alter their function from floral inducers to floral repressors, as demonstrated for sugarcane Coelho et al. and beet (Pin et al., 2010). We propose that selecting for *FT/TFL1* sequence variations in crop plants may pave the way to further improvements in elite crop productivity.

## Author contributions

All authors listed, have made substantial, direct and intellectual contribution to the work, and approved it for publication.

## Funding

We gratefully acknowledge funding by the German Research Foundation (Priority Program 1530 and Deutsche Forschungsgemeinschaft).

### Conflict of interest statement

The authors declare that the research was conducted in the absence of any commercial or financial relationships that could be construed as a potential conflict of interest.
